# The international HAE guideline under real-life conditions: From possibilities to limits in daily life – current real-world data of 8 German angioedema centers 

**DOI:** 10.5414/ALX02530E

**Published:** 2024-11-14

**Authors:** Jens Greve, Robin Lochbaum, Susanne Trainotti, Eva-Vanessa Ebert, Thomas Buttgereit, Antonia Scherer, Lisa Knipps, Anna Smola, Sebastian Volc, Andreas Recke, Katharina Marlies Duda, Mathias Sulk, Janina Hahn

**Affiliations:** 1 Department of Oto-Rhino-Laryngology. Head and Neck Surgery, Ulm University Medical Center, Ulm; 2 Department of Otorhinolaryngology, Technical University of Munich, Munich,; 3 Institute of Allergology, Charité-Universitätsmedizin Berlin, Corporate Member of Freie Universität Berlin and Humboldt-Universität zu Berlin, Berlin,; 4 Department of Otorhinolaryngology, Universitätsklinikum Düsseldorf, Düsseldorf,; 5 Department of Oto-Rhino-Laryngology – Head and Neck Surgery, St.-Josefs-Hospital Hagen, University of Witten/Herdecke, Hagen,; 6 Department of Dermatology, University Hospital Düsseldorf, Medical Faculty, Heinrich-Heine-University Düsseldorf, Düsseldorf,; 7 Department of Dermatology, University of Tübingen, Tübingen,; 8 Department of Dermatology, Allergology and Venereology, University of Lübeck, Lübeck,; 9 Department of Dermatology and Allergy, Hannover Medical School, Hannover, and; 10 Department of Dermatology, University of Münster, Münster, Germany

**Keywords:** angioedema, guideline, bradykinin, hereditary, therapy, C1INH, acquired angioedema, children

## Abstract

Background and objectives: Patients with rare diseases like hereditary angioedema (HAE) are usually referred to an angioedema center to ensure guideline-compliant and experience-based therapy. Even though there are established guidelines and several approved therapeutics, there are still open questions and situations in the daily care of HAE patients, where an exchange between centers is needed. Materials and methods: A survey was conducted among physicians from German angioedema centers regarding challenges and issues in everyday HAE treatment. The main focus was on the topic of long-term prophylaxis (LTP). For rarer subcategories of angioedema, the centers conducted a literature review to discuss open questions. Results: The responses of 12 physicians from 8 angioedema centers were analyzed in the survey. The attack frequency was the most important criterion for deciding to initiate LTP in HAE patients (100%). Two centers no longer generally recommend the initiation of pre-interventional prophylaxis in HAE patients under LTP. The therapeutic concepts of acquired angioedema due to C1 inhibitor deficiency and HAE in children were two associated specialized areas that were discussed in more detail. Conclusion: The current guideline serves as the foundation for daily practice in treating HAE at specialized centers. Thus, for rare conditions like HAE, an exchange among the treating centers is essential to adequately address specific issues and rare subgroups.

## Introduction 

Hereditary angioedema (HAE) is a rare genetic disease. The prevalence of HAE due to C1 inhibitor (C1INH; HAE-C1INH) deficiency is estimated to vary between 1.1 and 1.6 per 100,000 [1]. 

A lot of progress could be achieved during the last decades regarding the knowledge and therapeutic options of HAE and associated diseases like acquired angioedema due to C1INH deficiency (AAE-C1INH): Since the end of the 1970s, publications on effective on-demand therapy with C1INH have emerged. Overall, knowledge about the pathomechanisms in HAE has gradually expanded during this time. Based on quantitative and functional findings, two types of HAE were defined and diagnosed. Until 1979, ~ 15 cases of acquired C1INH deficiency with angioedema were documented. The clinical syndrome of angioedema in these patients resembled HAE, with low C1INH levels and C4 complement levels. However, instead of a genetic disorder, these patients typically had an underlying lymphoproliferative or autoimmune disease. It was already known that the primary goal of treatment was the therapy of the underlying disorder, but drugs effective in HAE (like androgens) were discussed to be helpful in preventing edema, also in AAE-C1INH. 

Studies conducted by Shoemaker et al. [2] further analyzed the pathomechanism in HAE. They demonstrated that bradykinin is the mediator of enhanced vascular permeability in the formation of angioedema. Intravenous administration of C1INH was not only effective in treating acute attacks but also for short-term prophylaxis (STP) before medical interventions. Different mutations in the “C1INH gene” *SERPING1* have been found and published as causes of HAE since the 1990s [3]. Attenuated androgens were known to effectively prevent attacks in HAE; nevertheless, concerns about their long-term safety arose. In 2000, cases of a new “estrogen-dependent inherited form of angioedema” were described for the first time. It was found that this type did not result from C1INH deficiency or dysfunction, but the clinical picture resembled HAE in many ways [4]. 

In 2002, a review on the management of HAE patients stated that “the indication for long-term prophylaxis (LTP) should be considered in each individual, but it is necessary to devise a regimen for each affected individual guided by the severity of the disease”. Frequent attacks of peripheral angioedema (such as in the extremities and abdomen) were considered unpleasant and annoying but were not deemed to require LTP as they did not pose a vital threat to the patients [5]. In 2004, proceedings of the Third C1INH Deficiency Workshop was published. LTP was pointed out for HAE patients experiencing many harmful and disturbing angioedema attacks, typically those with more than 1 attack per month. Around the same time, the bradykinin type 2 receptor antagonist icatibant (Hoe140) was being investigated for the treatment of acute HAE attacks. An analysis of self-administration of HAE-specific drugs was conducted to achieve rapid and more effective treatment. In 2006, missense mutations in the coagulation factor XII gene were discovered in patients with HAE with normal C1INH values [6]. In 2007, results were published on the effectiveness of ecallantide, a novel kallikrein inhibitor, for HAE treatment. In recent years, further progress has been made regarding the knowledge of other mutations leading to HAE with normal C1INH values (HAE-nC1INH). C1INH administered subcutaneously was licensed for LTP in HAE, as were the kallikrein inhibitor lanadelumab and the first oral treatment option berotralstat (also an inhibitor of plasma kallikrein). One of the most recent changes in HAE management was the consensus of the “International WAO/EAACI Guideline for the Management of HAE – The 2021 Revision and Update” regarding the indication for LTP in HAE patients [7]: The goals of treatment are to achieve total control of the disease and normalize patients’ lives; LTP should be individualized and considered in all HAE patients, taking into consideration disease activity, patient’s quality of life, availability of healthcare resources, and failure to achieve adequate control by appropriate on-demand therapy. 

### Objectives 

The well-established and detailed national and international HAE guidelines provide an excellent foundation for the day-to-day treatment of HAE patients [8]. However, HAE is an individual disease, requiring many decisions to be made on a patient-specific basis or often through shared decision-making. Additionally, there are associated, even rarer diseases, such as HAE-nC1INH and AAE-C1INH, which are not the primary focus of the guidelines but nonetheless present highly debilitating and even life-threatening clinical scenarios that must be treated accordingly. The aim of this manuscript is to address and discuss open questions and problems from the “real-world” setting with angioedema patients in a multicenter publication. 

## Materials and methods 

A questionnaire survey among German HAE centers, focusing on LTP in HAE patients, was conducted. Second, in a joint meeting, associated angioedema subgroups were developed and discussed to address open questions regarding diagnosis and treatment. 

The questionnaire-based survey was distributed to all participating HAE centers in Germany, and the original German-language questionnaire is available in the Supplemental Material. The survey focused on LTP from the therapist‘s perspective, inquiring about the indication criteria and the proportion of LTP patients relative to all HAE patients, followed by a subgroup analysis. The use of STP and the approach to patients with HAE-nC1INH were also investigated. 

Two validated questionnaires, regularly applied in all centers, recommended in the guidelines, and influencing the evaluation of results, will be briefly introduced: The Angioedema Quality of Life Questionnaire (AE-QoL) was employed to evaluate the quality of life of HAE patients, and the Angioedema Control Test (AECT) was used to assess disease control. 

The AE-QoL addresses the following four domains: functioning, fatigue/mood, fears/shame, and food. It is used to record the quality of life and its change over a period of time or under LTP. The questionnaire consists of 17 questions, where each answer represents a score between 0 and 4 points (maximum of all questions: 68 points). The total score is usually converted into a quality-of-life scale from 0 to 100%. The total score indicates the extent to which quality of life is affected. The higher the score, the lower the quality of life [9]. 

The AECT is based on 4 questions with recall periods of 4 weeks and 3 months. Each answer represents a score of between 0 and 4 points. A maximum of 16 points is possible and means complete control, whereas 0 points means no control at all. Patients with at least 10 points usually have adequate disease control [10]. 

At a joint meeting of the centers, three topics associated with HAE were identified where questions remain unanswered in daily practice, or due to the rarity of the disease. Some of them were already part of the survey of the centers. The three topics are listed below. For each of these topics, a brief overview has been prepared to present the open questions, discuss them, and recommend approaches based on an up-to-date literature review combined with the centers’ experiences.

- AAE-C1INH
- HAE-nC1INH
- HAE in children and adolescents

## Results 

### Survey of the centers with a focus on LTP 

12 angioedema experts (6 dermatologists, 5 otolaryngologists, and 1 internist) from 8 HAE centers throughout Germany participated in the survey. 

**Indication for LTP**

**Figure 1.**
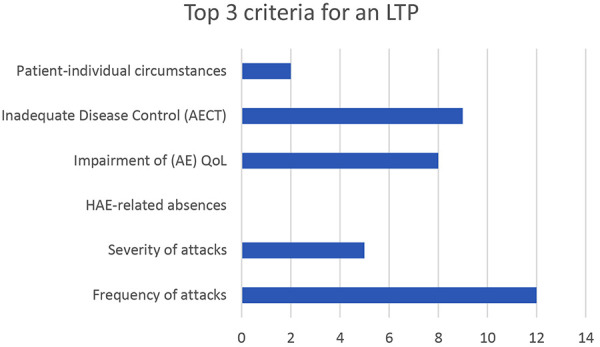
The most important criteria for indicating long-term prophylaxis (LTP) in patients with hereditary angioedema (HAE). Every expert was asked for his/her top 3 criteria. The question was answered by 12 experts from 8 angioedema centers.

When determining the indication for LTP, the 3 most important criteria assessed were “attack frequency” (12/12, 100%), „inadequate disease control“ evaluated using the AECT (9/12, 75%), and “impairment of quality of life” evaluated using the AE-QoL questionnaire (8/12, 66.7%) (Figure 1). 

An attack frequency of more than 6 attacks annually was considered an indication for LTP by 75% of the physicians, whereas for 25%, the threshold was higher than 12 attacks per year. Concerning quality of life, 83.3% of the respondents identified an LTP indication at 24 points on the AE-QoL (small effect on quality of life), while 16.7% considered more than 38 points (moderate to large effect on quality of life) as the threshold. For disease control, measured using AECT, all participants noted an LTP indication below 10 points. Additionally, 5 physicians mentioned the severity of HAE attacks as a criterion for prophylaxis, and 2 doctors identified patient-specific circumstances as an indication. 

The percentage of HAE patients who received LTP treatment in the centers was an average of 22.3% (± 21.4) by the end of 2018, increasing to 41.5% (± 17.9) by the beginning of 2022 (at the point of the survey). 

**Figure 2.**
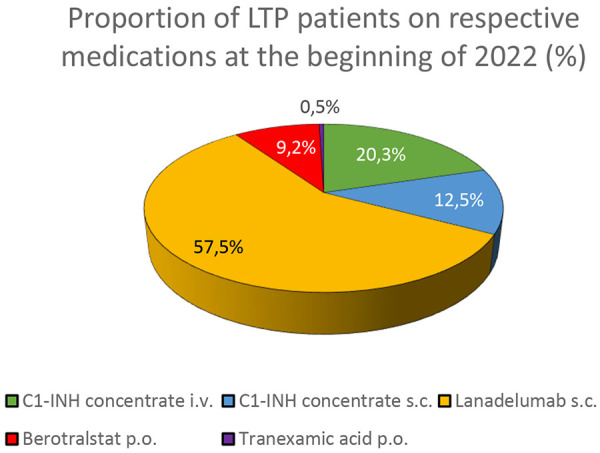
The proportion of long-term prophylactic treatment medications in patients with hereditary angioedema at the beginning of 2022. The question was answered by 12 experts from 8 angioedema centers.

At the start of 2022, patients receiving prophylactic treatment in the centers were separated by medication type as follows: 20.3% (± 15.9) of the patients received C1INH concentrate intravenously, 12.5% (± 12.0) C1INH concentrate subcutaneously, 57.5% (± 22.0) lanadelumab, 9.2% (± 6.3) berotralstat, and 0.5% (± 0.9) tranexamic acid (Figure 2). 

The proportion of children under 12 years of age in the LTP population of the centers is less than 1% (0.68% ± 1.1), all of these children receive C1INH concentrate intravenously for prophylaxis. (Note: lanadelumab was not yet approved for children < 12 years at the time of the survey). 

**STP in HAE patients with LTP**

2/12 physicians no longer explicitly recommend STP to their patients taking regularly and effective LTP. 10/12 doctors recommend STP prior to general anesthesia, 5/12 prior to dental treatments, 5/12 before outpatient procedures, and 4/12 before giving birth. 

**Further course of disease under LTP**

The optimization of LTP in the centers primarily relies on personal consultations with the patient (9/12) and the combination of AE-QoL and AECT scores (8/12). Furthermore, 5/12 surveyed doctors utilize the therapy records stored in patient files as a reference when optimizing therapy. 

**Figure 3.**
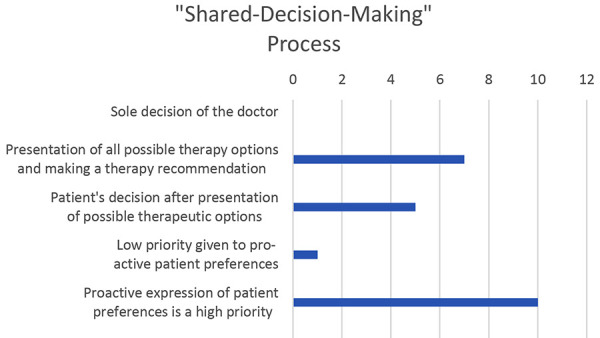
The most important steps of the shared-decision-making process. The question was answered by 12 experts from 8 angioedema centers. Multiple answers were possible.

In the decision-making process regarding LTP, shared decision making is of high significance to all colleagues (Figure 3): of the 12 physicians, 10 prioritize the proactive expression of the patient‘s preference, 7 provide all possible therapeutic options to the patient and then make a recommendation, while 5 allow the patient to decide after presenting them with the possible therapeutic options. One surveyed doctor deprioritizes the proactively expressed patient preference. 

11/12 doctors surveyed present new therapeutic options to patients during individual consultations in a balanced manner. Seven of them emphasize the relevant therapeutic features, while the remaining 5 discuss the efficacy and tolerability profile. Additionally, 4 colleagues provide informational materials for patients to take away. 

**Figure 4.**
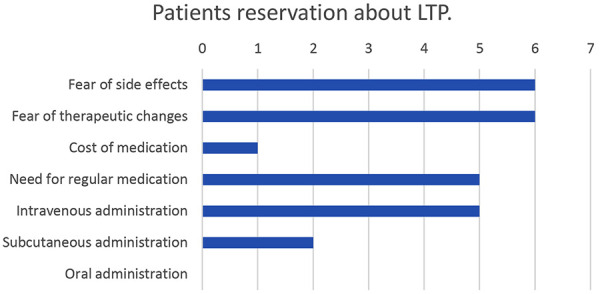
Patients’ most frequent reservations about long-term prophylaxis, reported by 12 experts from 8 angioedema centers. Multiple answers were possible.

At the start of 2022, the centers had an average of 20% (± 12.6%) of HAE patients who, from the perspective of the treating physicians, were recommended LTP but refused the treatment at that point. The reasons, why the physicians surveyed had not yet adapted patients who medically required LTP to LTP, were patient reservations about LTP in 9/12 cases, poor patient accessibility in 5/12, and lack of patient compliance in 4/12 cases. Patient reservations about LTP mentioned by physicians were “fear of side effects” in 6 and “fear of therapeutic changes” in another 6. 5/12 physicians mentioned the “need for regular medication” and an “intravenous form of administration”. The “subcutaneous route” (2/12) and “cost of medication” (1/12) played a minor role. The patients’ reservations about LTP are summarized in Figure 4. 

**Treatment of HAE-nC1INH**

**Figure 5.**
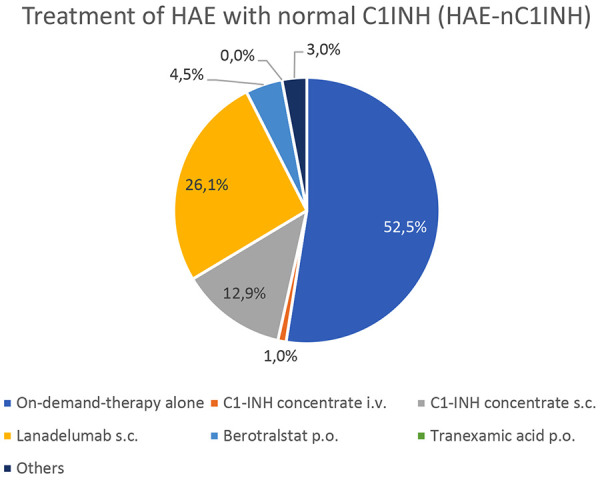
The treatment of the patients with hereditary angioedema (HAE) with normal C1 inhibitor (C1INH), reported by 12 experts from 8 angioedema centers. (Note: the treatment of HAE with normal C1INH is a grey area, as the drugs are only licensed for HAE-C1INH).

The treatment of HAE-nC1INH was also questioned and discussed among the centers (Figure 5). In 52.5% of cases, only on-demand therapy is used. Additional kallikrein inhibitors as LTP, specifically lanadelumab (26.1%) and berotralstat (4.5%), are the second most common treatment. C1INH is administered subcutaneously in 12.9% and intravenously in 1% of cases. Antifibrinolytic therapy, such as tranexamic acid, is not used by the surveyed practitioners; however, other (not further specified) medication is used in 3% of cases. Treatment choices vary based on individual factors and the severity of the condition. 

### HAE-associated conditions: AAE, HAE-nC1INH, and HAE in children and adolescents 

**AAE-C1INH**

AAE-C1INH is ~ 10 times rarer than HAE. Similar to HAE due to C1INH deficiency, patients suffer from recurrent swellings of the face and abdomen, however they first occur in adulthood at the age of 40 and beyond and with a negative family history [11]. In contrast to HAE, the symptoms are not caused by problems in the production of C1INH, but due to cleavage through antibodies or direct consumption of C1INH. Furthermore, unlike HAE-C1INH, the face is the most frequently affected location. 

Blood tests show low levels of C1INH antigen, C1INH function, and C4, as well as low C1q (about 70% of patients) – the latter is extraordinary rare for HAE-C1INH patients. If still in doubt, genetic testing for *SERPING1* (encoding C1INH) can be performed. 

Common underlying diseases are hemato-oncological conditions, e.g., indolent B-cell-lymphomas or monoclonal gammopathies of undetermined significance (MGUS) or autoimmune diseases [11], but also solid tumors [12]. As a consequence, it is crucial to try to detect the underlying – often malignant – disease. In these cases, treatment of the primary disease is the only recommended approach. Unfortunately, AAE-C1INH not always resolves afterwards and may recur. Furthermore, in many cases no underlying disease can be identified, but also spontaneous resolution is possible. 

Since no controlled clinical trials have been done due the low number of patients for this extremely rare condition, pharmacologic treatment of AAE-C1INH is off-label and follows the principles for HAE treatment. Good effectiveness is reported by several studies for the following drugs: possible on-demand treatment includes the replacement of intravenous C1INH, which can also be used as STP, as well as the inhibition of the bradykinin B2 receptor with subcutaneous icatibant. For LTP, the three drugs available for HAE in Europe show also good efficacy for AAE-C1INH in case studies: for subcutaneous application, C1INH concentrate or lanadelumab can be administered. Additionally, the oral LTP berotralstat (small molecule plasma kallikrein inhibitor) is available. 

Rituximab is described as an effective therapy for AAE-C1INH patients with, but also without, lymphoproliferative diseases or MGUS in several series. Due to the potentially immunosuppressive side effects of rituximab, the decision for treatment should be made in close dialogue with the patient, especially in patients without underlying lymphoproliferative diseases. Since AAE-C1INH patients are a heterogeneous group with different underlying conditions showing the same phenotype, the first step should be to identify and, if possible, treat the underlying disease. If this is not possible or insufficient, patients should be given the opportunity to treat the swellings in analogy to patients with HAE according to WAO/EAACI guidelines, even though it is an off-label use [13]. 

**HAE-nC1INH**

So far, HAE-nC1INH (formerly HAE type III) has been described as relatively rare, with definitive statements difficult due to the lack of clear diagnostic criteria. Initial prevalence surveys suggest that this form of HAE may be more common than initially thought. 

In contrast to HAE-C1INH, there is no specific laboratory test for confirming the diagnosis of HAE-nC1INH. Genetic testing is often inconclusive; 8 mutations are so far known to cause HAE-nC1INH (aactor XII, angiopoietin-1, plasminogen, kininogen-1, myoferlin, heparan sulfate 3-O-sulfotransferase 6, carboxypeptidase N, and DAB2IP). Many of them have only recently been identified, thus more yet unknown mutations can be assumed. Diagnosis usually is based on the patient’s family history, clinical presentation, and response to various therapeutic attempts [14], if genetic testing is inconclusive. 

Nonetheless, diagnostic uncertainty is always present in HAE-nC1INH without a detected mutation. The most challenging differential diagnosis of HAE-nC1INH without mutation is certainly chronic spontaneous urticaria (CSU) without wheals. One fact to consider in general is the incidence of the diseases: CSU is a lot more frequent than HAE-nC1INH. 

Another possibility to try to differentiate between the two conditions is the response to treatment, although this may not be true in all patients. The commonly cited lack of response to corticosteroids, antihistamines, or adrenaline in emergency situations is often not a very reliable criterion in practice. The response to prophylactic therapy for CSU can be assessed first: a systematic treatment with antihistamines in an up to quadrupled standard dose (off-label, according to the current urticaria guideline), followed by omalizumab add-on if patients are still symptomatic. This approach requires good patient management. Therefore, it is important to develop new biomarkers to aid in the diagnosis of HAE-nC1INH [15]. 

A peculiarity of the treatment options in HAE-C1INH like kallikrein inhibitors as well as tranexamic acid is that they have only been tested in studies involving HAE-C1INH; details are summarized in Table 1. In addition to bradykinin, other mediators like VEGF-C have been described in forms of HAE-nC1INH like HAE with angiopoietin-1 and myoferlin mutations. No effective therapies have been identified for these HAE variants yet. 

**HAE in children and adolescents**

The management of HAE-C1INH in children is addressed in a separate section of the current guideline [7]. In principle, HAE is a rare disease for which very well-established therapies are available for children. However, the diagnostic management and initiation of therapy in children poses challenges. The first symptoms of HAE can manifest at any age, but the majority of patients (~ 90%) present before the age of 20. One difficulty is that abdominal attacks in children are often not attributed to HAE as children often present with abdominal symptoms due to other more frequent conditions. 

One point of discussion is the timepoint of testing: according to the current international HAE guideline, children of parents with HAE-C1INH should be tested as early as possible [7]. In this way, treatment can be tailored to the needs of the child and the first acute episodes of swelling can be treated quickly and appropriately. Laboratory diagnostics include complement measurement and genetic testing, as in adults. However, it is important to note that before the age of 1 year, C1INH levels are sometimes lower than in adults, with the lowest levels found in umbilical cord blood. Therefore, if in doubt, retesting after the first year of life is recommended, and the interpretation of cord blood sampling should be undertaken with extreme caution. 

Questionnaires such as the AECT and the AE-QoL to assess disease control in patients with recurrent angioedema have only been validated in adults (> 18 years) [10]. The Pediatric Quality of Life Inventory (PedsQL) was used to assess health-related quality of life (HRQoL) in children by Engel-Yeger et al. [16]. However, HRQoL in children with HAE-C1INH is almost unexplored. The PedsQL (Child Self-Report and Parent Proxy-Report forms), the Children‘s Dermatology Life Quality Index, an unvalidated, disease-specific quality of life questionnaire, and two visual analog scales to assess overall health can be used. Especially with young children, the medical history or interview regarding the current disease burden and discussion of the therapy regimen should be conducted with both the patient and the caregivers. The management of HAE in children and adolescents requires a differentiated approach for optimal treatment. Involvement of parents and children in decision-making plays a central role, not only in increasing the effectiveness of the therapy but also in improving the quality of life of affected children and adolescents. 

The impact of HAE attacks on school attendance and academic performance may affect future career or educational opportunities. The results of recent studies show a higher prevalence of HAE symptoms in children under the age of 12 years compared to previous data. Particular attention should be paid to the psychological aspects of HAE. 

A recent study demonstrated the safety, efficacy, and improved quality of life through LTP with lanadelumab to prevent angioedema attacks in children aged 2 to under 12 years [17]. Table 1 summarizes the current situation of HAE treatment options in children and adolescents. 

## Discussion 

In the questionnaire-based survey, the authors looked closely at healthcare providers` treatment decisions regarding LTP. It became evident that, for many providers, personal discussions with their patients combined with patient-reported outcome measures (PROMs), especially AE-QoL and AECT, constituted the primary basis for decisions. Additionally, individual disease courses and experiences with previous treatment approaches were considered. When asked why some patients had not been prescribed LTP so far, reservations about new therapies were predominantly cited as the main reason. Some patients were challenging to reach or demonstrated poor compliance. 

Interestingly, there was no clear trend in the preferred mode of administration between subcutaneous and oral, while intravenous administration was favored by only a few. Looking at the reasons for discontinuation or a change in LTP, it was evident that side effects, low effectiveness, and inadequate patient satisfaction were the primary reasons. Another important reason was the need for a change due to family planning and pregnancy in female patients. C1INH substitution is the only therapy considered a safe option in case of family planning or pregnancy [7]. 

Regarding the methods employed by healthcare providers in offering LTP to their patients, the investigation revealed that most providers present and explain all available treatment options. In conclusion, it was evident that when presenting treatment options, all providers strive to ensure a balanced representation. Some focus more on the efficacy and tolerability profiles, supplementing their discussions with informational materials. Not yet discussed were approaches to CRISPR-Cas-based gene therapy with a single – and not regular – application of the medication. This therapy is currently in phase I/II studies, and its future role remains to be seen. 

In 2020, an estimated 28% of HAE patients in Germany received LTP. According to a Delphi consensus from 8 German HAE centers, it was estimated that the rate of LTP patients will increase to 40% in 2023 [18]. This assumption supports the requirement of the WAO/EAACI guideline that the goal of HAE treatment should not only be the reduction of attacks but also an increase in quality of life as far as normalizing patients’ lives [7]. Patients not only suffer from their disease during an acute attack but also in phases in between due to the unpredictability of these attacks, which results in a massive reduction of quality of life. To fulfil the requirement of the guideline, it should be discussed whether every HAE patient has the right to be offered LTP. Advances in LTP offer the opportunity to treat patients individually with focus on their quality of life rather than looking at attack frequency and severity. The authors would like to stress that the above-mentioned considerations are only possible due to the current favorable LTP availability in Germany and thus might not easily be transferred to other countries’ situations. 

An important topic of the questionnaire was whether all HAE patients under LTP still need to be recommended pre-interventional prophylaxis. So far, there are not many data on this. Multiple prior studies have shown that prophylaxis before medical or dental procedures significantly reduces the postoperative attack rate in HAE patients. According to the WAO guideline it is suggested “considering prophylaxis prior to exposure to patient-specific angioedema-inducing situations” [7]. Especially if a patient has had HAE symptoms after a certain procedure, this procedure should be considered as a high risk for the patient. However, in the authors’ clinical experience, well-controlled modern LTP patients use less STP peri-procedurally (without prior consultation with the treating physicians) and report usually no increased attack rates. STP treatment plans should be personalized based on the respective medical or dental procedures, available HAE therapies, and on shared decision making with the patient or caregivers [19]. With the currently available data, a unified recommendation cannot yet be given to forgo STP in HAE patients under LTP. 

Due to the high costs of prophylaxis and the expected increasing number of patients under LTP (as shown in the questionnaire evaluation), the discussion about extending intervals for symptom-free HAE patients under LTP is inevitable. In the scientific literature, there are few studies or recommendations regarding the approach to extending the dosing interval of LTP. This may be due, in part, to the fact that extending the dosing interval is off-label for some first-line recommended therapy options, and for others, a reduction in effectiveness may be expected. The most data on dose interval extension exist for lanadelumab. The recommended initial dose is 300 mg every 2 weeks. According to the product information, for patients who are stable and attack-free under LTP with lanadelumab, a dose reduction to 300 mg every 4 weeks may be considered, especially for those with low body weight. Based on practical experiences demonstrating the high effectiveness of lanadelumab, it has been observed that many patients remain attack-free with injections every 2 weeks and choose to administer the therapy at 4-week intervals. In ACARE Berlin, a potential concept for identifying the optimal dosing interval for lanadelumab was developed. This involves a gradual increase of the interval by 3 days starting from the 3^rd^ administration in attack-free patients [20]. If prodromal signs or breakthrough attacks occur during the dose interval extension, the interval is reset to the duration of the penultimate dosing interval. This approach has proven effective in a described patient population, simultaneously reducing the burden of treatment over a long period of time. 

Due to the frequently occurring side effects and the relatively high monitoring effort compared to first-line LTP recommendations, androgens are only recommended as a second-line therapy in many countries such as Germany. The results of the STAR-Network survey indicate that androgens are no longer prescribed as LTP in German angioedema centers. Nevertheless, in some countries, androgens have been used as LTP for a long time or continue to be prescribed due to the unavailability of first-line recommendations. Currently, there is insufficient experience to provide recommendations on how androgens should be discontinued, i.e., either abruptly or gradually. A gradual discontinuation approach may possibly mitigate the risks of increased frequency and severity of attacks, as well as the occurrence of side effects (e.g., fatigue, mood swings, anxiety and depression, and sexual dysfunction). Future studies, such as the ACARE project SHAERPA (https://acare-network.com/project/shaerpa/), should investigate in more detail the optimal methods for initiating and discontinuing androgens. 

## Conclusion 

The course of HAE varies from patient to patient. As the disease itself is a genetic disorder and current treatments cannot cure the gene mutation, patients will suffer from the risk of asphyxiation – regardless of the previous attack rate. Therefore, also asymptomatic and low-attack patients can experience anxiety. However, as the disease severity can vary over time, the need for LTP should be regularly reviewed together with the patient. It is recommended to assess the disease activity and quality of life in HAE patients using validated tools. Uncertainty in the diagnosis is always present in HAE-nC1INH without detected mutation. Usually in these patients as well as in AAE-C1INH patients, the therapeutic regimen is based on the approach for HAE as guidelines usually focus on HAE-C1INH. Especially in young children with HAE, the medical history or interview regarding the current disease burden and discussion of the therapy regimen should be conducted with both the patient and the caregivers. 

## Authors’ contributions 

JH and JG conceived of the presented idea and supervised and organised the project. All authors contributed to the introduction and discussion. JH, JG and RL developped the questionnaire with the help of all authors. KMD and JH focused on HAE in children and adolescents, ST and EVE focused on Aquired Angiooedema, AS, LK and ASm focused on STP and LTP, TB and MS focused on special questions concerning LTP (like androgens), AR and SV focused on shared decision making and HAE-nC1INH. All authors provided critical feedback and helped shape the research, analysis and manuscript. 

## Funding 

We thank the HAE medical team from Takeda Pharma Vertrieb GmbH & Co, KG for organizing regular virtual group meetings. Takeda had no role in the selection of experts or the collection, analysis, and interpretation of the data. The publication was set up and written independently by the authors. No funding was received.

## Conflict of interest 

The multicenter project received support from Takeda Pharmaceuticals. 

J. Greve: has received speaker/consultancy fees from CSL Behring, Kalvista Takeda, and BioCryst. He has also received funding to attend conferences/educational events from CSL Behring and Takeda. He has participated as an investigator in a clinical trial/registry for CSL Behring, Pharvaris, BioCryst and Takeda. 

R. Lochbaum: has received funding to attend conferences/educational events from CSL Behring, BioCryst, and Takeda. He has participated as an investigator in a clinical trial for Takeda and Pharvaris and received financial support for research projects from Takeda. 

S. Trainotti: has received grant research support and/or speaker/consultancy fees from CSL Behring and Takeda. She has also received funding to attend conferences/educational events from CSL Behring and Takeda. She has participated as an investigator in a clinical trial/registry for CSL Behring, BioCryst, IONIS Pharmaceuticals, and Takeda. 

E.-V. Ebert: has received grant research support and/or speaker/consultancy fees from CSL Behring and Takeda. She has also received funding to attend conferences/educational events from CSL Behring, Takeda, and Pharming. She has participated as an investigator in a clinical trial/registry for CSL Behring, BioCryst, IONIS Pharmaceuticals, and Takeda. 

T. Buttgereit: was a speaker, and/or advisor for, and/or has received research funding from Aquestive, BioCryst, CSL Behring, GSK, Hexal, KalVista, Medac, Novartis, Pharming, Roche, Sanofi-Aventis, Swixx BioPharma, and Takeda. 

A. Scherer: has received speaker/consultancy fees from CSL Behring, BioCryst, and Takeda. She has also received funding to attend conferences/educational events from CSL Behring and Takeda. She has participated as an investigator in a clinical trial/registry for CSL Behring and Takeda. 

L. Knipps: has received speaker/consultancy fees from CSL Behring, BioCryst, and Takeda. She has also received funding to attend conferences/educational events from CSL Behring and Takeda. She has participated as an investigator in a clinical trial/registry for Takeda. 

A. Smola: has received speaker/consultancy fees from Takeda and BioCryst. She has also received funding to attend conferences/educational events from CSL Behring and Takeda. 

S. Volc: has received speaker/consultancy fees from Takeda. He has participated as an investigator in a clinical trial/registry for Takeda. 

A. Recke: has received research grants from Deutsche Forschungsgemeinschaft and Euroimmun; other research support from CSL Behring, Novartis, Pharming, Stallergenes Greer, and Takeda; honoraria and/or travel grants from BENCARD, BioCryst, CSL Behring, Euroimmun, Novartis, and Takeda; and served as a consultant or participated in advisory boards for BioCryst, CSL Behring, Novartis, Swedish Orphan Biovitrum, and Takeda. 

K.M. Duda: has received funding to attend conferences/educational events from CSL Behring, BioCryst, and Takeda. She has participated as a sub-investigator in a clinical trial/registry for Takeda and she has received speaker/consultancy fees from CSL Behring, BioCryst, and Takeda. 

M. Sulk: was a speaker, and/or advisor for, and/or has received research funding from AstraZeneca, Bencard, BioCryst, CSL Behring, HAL Allergie, LEO Pharma, Novartis, RG Gesellschaft für Information und Organisation, Sanofi, Takeda, Unna Akademie. 

J. Hahn: has received speaker/consultancy fees from CSL Behring, BioCryst, and Takeda. She has also received funding to attend conferences/educational events from CSL Behring and Takeda. She has participated as an investigator in a clinical trial/registry for CSL Behring, BioCryst, Pharvaris, and Takeda. 

**Table 1.** HAE treatment options in children and adolescents.

**Approved**					
**Active substance**	**Mode of action**	**Brand name**	**Approved for (age)**	**Indication**	**Used for**
Berotralstat (oral)	Plasmakallikrein inhibitor	Orladeyo	12 years	HAE wR	LTP
Danazol (oral)*	Androgen	Danatrol is no longer manufactured in G, Danokrin from A is available	18 years	HAE wR	LTP
Icatibant SC	Bradykinin receptor 2 antagonist	Firazyr	2 years	HAE-C1INH	AT
Lanadelumab SC	Plasmakallikrein inhibitor	Takhzyro	2 years	HAE wR	LTP
pdC1INH IV	pdC1INH	Cinryze	2 years	HAE wR	STP, LTP
pdC1INH IV	pdC1INH	Berinert 500/1,500	wR	HAE-C1INH	AT, STP, LTP
pdC1INH SC	pdC1INH	Berinert 2,000/3,000	wR	HAE-C1INH	STP, LTP
Tranexamic acid (oral)	Antifibrinolytic	Cyclokapron	wR	HAE wR	LTP
**In clinical trials (according to ClinicalTrials.gov)**					
**Active substance**	**Mode of action**	**Sponsor**	**Age (min.)**	**Tested indication**	**Phase**
Donidalorsen s.c.	Plasmakallikrein inhibitor	Ionis Pharmaceuticals	12 years	HAE-C1INH	3
Garadacimab s.c.	Faktor XIIa inhibitor	CSL Behring	12 years	HAE-C1INH	3
KVD900 p.o.	Plasmakallikrein inhibitor	KalVista Pharmaceuticals	12 years	HAE-C1INH	3
PHA121 p.o.	Bradykinin receptor 2 antagonist	Pharvaris	18 years	HAE-C1INH	2/3
NTLA-2002	CRISPR-Cas knockout of *KLKB1* (Prekallikrein)	Intellia	18 years	HAE-C1INH	1/2
STAR-0215	Long actin Kallikrein inhibitor	Astria Therapeutics	18 years	HAE-C1INH	1/2
ADX-324	siRNA knockdown *KLKB1*	ADARx Pharmaceuticals	18 years	HAE-C1INH	1/2
GNR-038	Recombinant C1INH	Russian federation	18 years	HAE-C1INH	1/2

A = Austria; AT = acute therapy; G = Germany; HAE = hereditary angiodema; LTP = long term prophylaxis; pdC1INH = plasma-derived C1-esterase inhibitor; STP = short term prophylaxis; TBA = to be announced; wR = without restriction. *Recommended as second-line long-term prophylaxis only. All information (with the exception of Danazol) was taken from the product information, www.fachinfo.de, accessed September 24, 2023; Information about Danazol accessed from https://medikamio.com/de-at/medikamente/danokrin-200-mg-kapseln/pil, September 24, 2023.

## Supplemental material

Supplemental material German-language questionnaire
