# Supplementary material for: The international HAE guideline under real-life conditions: From possibilities to limits in daily life – current real-world data of 8 German angioedema centers
Source: Allergol Select. 2024 Nov 14;8:346–57. doi: 10.5414/ALX02530E (PMC11575681; doi:10.5414/ALX02530E)
Supplement: Supplemental material [file allergologieselect-8-346-S01.pdf]

# FRAGEBOGEN HAE LANGZEITPROPHYLAXE DEUTSCHLAND

## 1) Anhand welcher Kriterien stellen Sie die Indikation für eine Langzeitprophylaxe (Mehrfachnennung möglich)?

| | | | | | |
|---|---|---|---|---|---|
| **Frequenz der Attacken** | **Nein ☐** | **Ja ☐** | **Falls „ja“, Frequenz?** | **<6 / Jahr** | ☐ |
| | | | | **6 bis <12/ Jahr** | ☐ |
| | | | | **12 bis <24 / Jahr** | ☐ |
| | | | | **≥24 / Jahr** | ☐ |
| **Schwere der HAE Attacken** | **Nein ☐** | **Ja ☐** | **Falls „ja“, Ausprägung?** | **Kopf-Hals Beteiligung** | ☐ |
| | | | | **Starke Schmerzen** | ☐ |
| | | | | **Hospitalisierung** | ☐ |
| | | | | **Sonstiges (bitte angeben):** | |
| **HAE-bedingte Fehltage** | **Nein ☐** | **Ja ☐** | **Falls „ja“, Anzahl?** | **0 bis <5 / Monat** | ☐ |
| | | | | **5 bis <10 / Monat** | ☐ |
| | | | | **10 oder mehr / Monat** | ☐ |
| **Beeinträchtigung der Lebensqualität (AE-QoL)** | **Nein ☐** | **Ja ☐** | **Falls „ja“, Score?** | **0 bis 23 Punkte** | ☐ |
| | | | | **24 bis 38 Punkte** | ☐ |
| | | | | **>38 Punkte** | ☐ |
| **Unzureichende Krankheitskontrolle (AECT)**<br>Nein, falls Score > 10 Punkte<br>Ja, falls Score ≤ 10 Punkte | **Nein ☐** | **Ja ☐** | | | |
| **Patient*innen- individuelle Umstände** | **Nein ☐** | **Ja ☐** | **Falls „ja“, welche?** | **Erzieherische Verpflichtungen/ Fürsorge** | ☐ |
| | | | | **Soziale Verpflichtungen** | ☐ |
| | | | | **Berufliche Verpflichtungen** | ☐ |
| | | | | **Wunsch/Angst des Patienten** | ☐ |
| | | | | **Sonstige (bitte angeben):** | |

## 2) Welches sind Ihre <u>Top-3-Kriterien</u> für eine Langzeitprophylaxe? (bitte nur 3 auswählen)

| | |
|---|---|
| **Frequenz der Attacken** | ☐ |
| **Schwere der HAE Attacken** | ☐ |
| **HAE-bedingte Fehltage** | ☐ |
| **Beeinträchtigung der Lebensqualität (AE-QoL)** | ☐ |
| **Unzureichende Krankheitskontrolle (AECT)** | ☐ |
| **Patient*innen- individuelle Umstände** | ☐ |

| 3) Anteil an Langzeitprophylaxe-Patient*innen an Ihrer Klinik (bitte in Prozent angeben) | |
|---|---|
| **Wie hoch war dieser Ende 2018?** | % |
| **Wie hoch ist dieser aktuell?** | % |
| **Wie hoch erwarten Sie diesen Ende 2023?** | % |

| 4) Wie ist aktuell die Aufteilung Ihrer Langzeitprophylaxe-Patient*innen nach Therapieoptionen? (bitte in Prozent angeben) | |
|---|---|
| **C1-INH Substitution i.v.** | % |
| **C1-INH Substitution s.c.** | % |
| **Plasmakallikrein-Inhibitor (Antikörper) s.c.** | % |
| **Plasmakallikrein-Inhibitor (small molecule) p.o.** | % |
| **Antifibrinolytikum p.o.** | % |
| **Sonstige (bitte angeben):** | % |

| 5) Wie erwarten Sie Ende 2023 die Aufteilung Ihrer Langzeitprophylaxe-Patient*innen nach Therapieoptionen? (bitte in Prozent angeben) | |
|---|---|
| **C1-INH Substitution i.v.** | % |
| **C1-INH Substitution s.c.** | % |
| **Plasmakallikrein-Inhibitor (Antikörper) s.c.** | % |
| **Plasmakallikrein-Inhibitor (small molecule) p.o.** | % |
| **Antifibrinolytikum p.o.** | % |
| **Sonstige (bitte angeben):** | % |

| 6) Wie hoch ist aktuell der Anteil an Kindern (< 12 Jahren)? | |
|---|---|
| **In Ihrer gesamten Patientenpopulation?** | % |
| **In Ihrer Langzeitprophylaxe-Population?** | % |

| 7) Wie ist aktuell die Aufteilung Ihrer Langzeitprophylaxe-Kinder (< 12 Jahren) nach Therapieoptionen? (bitte in Prozent angeben) | |
|---|---|
| **C1-INH Substitution i.v.** | % |
| **Antifibrinolytikum p.o.** | % |
| **Sonstige (bitte angeben):** | % |

| 8) Nutzen Ihre derzeit (aus Ihrer Sicht) gut eingestellten Langzeitprophylaxe-Patient*innen noch zusätzlich prä-operative Kurzzeitprophylaxe? | | |
|---|---|---|
| **Bei Zahnbehandlungen** | Ja ☐ | Nein ☐ |
| **Bei Geburten** | Ja ☐ | Nein ☐ |
| **Bei ambulanten Eingriffen** | Ja ☐ | Nein ☐ |
| **Bei Operationen mit Vollnarkose** | Ja ☐ | Nein ☐ |
| **Sonstige (bitte angeben):** | Ja ☐ | Nein ☐ |

| 9) Wie überprüfen Sie Ihr Patient*innenkollektiv auf den potenziellen Bedarf einer Langzeitprophylaxe / Therapieoptimierung? (Mehrfachnennung möglich) | | | |
|---|---|---|---|
| Aus zeitlichen / organisatorischen Gründen ist im klinischen Alltag eine fortlaufende Prüfung oft nicht möglich | | | ☐ |
| Ich verlasse mich auf das persönliche Gespräch während der Visite | | | ☐ |
| Ich kontrolliere in regelmäßigen Abständen systematisch Patient*innenakten im Hinblick auf Therapieverläufe | | | ☐ |
| Ich nutze regelmäßig validierte Tools zur Lebensqualität & Krankheitskontrolle während der regulären Visiten | AE-QoL☐ | AECT☐ | AE-QoL + AECT ☐ |
| Ich nutze regelmäßig andere (validierte) Monitoring-Tools während der regulären Visiten | ☐ | Welche? (Bitte angeben): | |

| 10) Wieviel % Ihrer derzeit rein „on-demand“ behandelten Patient*innen sollten Ihrer Meinung nach eigentlich eine Langzeitprophylaxe erhalten? | |
|---|---|
| Bitte Prozentzahl angeben | % |

| 11) Aus welchen Gründen sind diese Patient*innen derzeit noch nicht auf eine Langzeitprophylaxe eingestellt? (Mehrfachnennung möglich) | |
|---|---|
| Patient*in hat eine schlechte Therapie-Compliance | ☐ |
| Patient*in ist schlecht erreichbar und erscheint nur unregelmäßig zu Visiten | ☐ |
| Patient*in hat konkrete Vorbehalte gegenüber einer Langzeitprophylaxe (s. Frage 12) | ☐ |
| Sonstige (bitte angeben): | |

| 12) Weshalb entscheiden sich Patient*innen Ihrer Meinung nach gegen eine Langzeitprophylaxe (Mehrfachnennung möglich)? | | | |
|---|---|---|---|
| Angst vor Nebenwirkungen | Nein ☐ | Ja ☐ | Falls „ja“, Welche? (Bitte angeben): |
| Angst vor therapeutischer Veränderung | Nein ☐ | Ja ☐ | |
| Preis der Medikamente | Nein ☐ | Ja ☐ | |
| Notwendigkeit der regelmäßigen Gabe | Nein ☐ | Ja ☐ | |
| Applikationsform i.v. | Nein ☐ | Ja ☐ | |
| Applikationsform s.c. | Nein ☐ | Ja ☐ | |
| Applikationsform p.o. | Nein ☐ | Ja ☐ | |
| Sonstige (bitte angeben): | | | |

| 13) Gab es in Ihrem Patientenkollektiv Abbrüche oder Umstellungen einer Langzeitprophylaxe? Falls ja, aus welchen Gründen? | | | |
|---|---|---|---|
| **Nebenwirkungen** | **Nein ☐** | **Ja ☐** | **Falls „ja“, Welche? (Bitte angeben):** |
| | | | **Falls „ja“, bei welchem Wirkstoff? (Bitte angeben):** |
| **Ungenügende Wirksamkeit** | **Nein ☐** | **Ja ☐** | **Falls „ja“, bei welchem Wirkstoff? (Bitte angeben):** |
| **Ungenügende Patientenzufriedenheit** | **Nein ☐** | **Ja ☐** | **Falls „ja“, bei welchem Wirkstoff? (Bitte angeben):** |
| **Ungenügende Patientencompliance** | **Nein ☐** | **Ja ☐** | **Falls „ja“, bei welchem Wirkstoff? (Bitte angeben):** |
| **Konkreter Patientenwunsch nach anderer Langzeitprophylaxe** | **Nein ☐** | **Ja ☐** | **Falls „ja“, bei welchem Wirkstoff? (Bitte angeben):** |
| **Schwangerschaft oder Kinderwunsch** | **Nein ☐** | **Ja ☐** | **Falls „ja“, bei welchem Wirkstoff? (Bitte angeben):** |
| **Umstellung auf ein anderes Präparat** | **Nein ☐** | **Ja ☐** | **Falls „ja“, auf welchem Wirkstoff? (Bitte angeben):** |

| 14) Weshalb entscheiden sich Patient*innen Ihrer Meinung nach <u>für</u> eine Langzeitprophylaxe? | | |
|---|---|---|
| **Gute Wirksamkeit & hohe Verträglichkeit** | **Nein ☐** | **Ja ☐** |
| **Einsparen von Akutmedikation** | **Nein ☐** | **Ja ☐** |
| **Individualisierbare Therapieoptionen** | **Nein ☐** | **Ja ☐** |
| **Sicherheit durch regelmäßige Verabreichung** | **Nein ☐** | **Ja ☐** |
| **Applikationsform i.v.** | **Nein ☐** | **Ja ☐** |
| **Applikationsform s.c.** | **Nein ☐** | **Ja ☐** |
| **Applikationsform p.o.** | **Nein ☐** | **Ja ☐** |
| **Sonstige (bitte angeben):** | | |

| 15) Inwieweit wenden Sie das Prinzip des „Shared Decision Making“ bei Umstellung auf eine Langzeitprophylaxe an? (Mehrfachnennung möglich) | |
|---|---|
| **Ich verlasse mich rein auf mein medizinisches Wissen und Einschätzungsvermögen** | ☐ |
| **Ich stelle Patient*innen im Gespräch alle Therapieoptionen vor, spreche aber eine Empfehlung aus** | ☐ |
| **Ich stelle Patient*innen im Gespräch alle Therapieoptionen vor und lasse sie am Ende selbst entscheiden** | ☐ |
| **Ein proaktiv geäußerter Patient*innenwunsch bezüglich einer Therapie hat für mich eine *geringe* Priorität** | ☐ |
| **Ein proaktiv geäußerter Patient*innenwunsch bezüglich einer Therapie hat für mich eine *hohe* Priorität** | ☐ |

| 16) Wie stellen Sie Ihren Patient*innen neue Therapieoptionen vor? (Mehrfachnennung möglich) | |
|---|---|
| **Ich stelle den Patient*innen im individuellen Gespräch alle Therapieoptionen ausgewogen vor** | ☐ |
| **Ich lege den Fokus im individuellen Gespräch in erster Linie auf das Wirksamkeits- & Verträglichkeitsprofil (z.B. aus Studiendaten)** | ☐ |
| **Ich lege den Fokus im individuellen Gespräch in erster Linie auf (aus meiner Sicht) Patient*innen-relevante Therapiemerkmale (z.B. Applikationsart)** | ☐ |
| **Ich gebe den Patient*innen Informationsmaterial zum Selbststudium mit nach Hause** | ☐ |
| **Ich habe kein einheitliches Vorgehen und sehe den Bedarf für eine neutrale Informationsplattform zu diesen Zwecken** | ☐ |

| 17) Greifen Sie auf die Möglichkeit zurück, Patient*innen bei der Langzeitprophylaxe durch einen spezialisierten Krankenpflege-Service/Health-Care-Service mitbetreuen zu lassen? | |
|---|---|
| **Ja, häufig** | ☐ |
| **Ja, in Einzelfällen** | ☐ |
| **Nein** | ☐ |

| 18) Betreuen Sie Patient*innen mit HAE mit normalem C1-INH? | |
|---|---|
| **Ja** | ☐ |
| **Nein** | ☐ |

| 19) Wenn Sie Frage 18 mit “Ja” beantwortet haben, welche Therapieoptionen verwenden Sie bei HAE mit normalem C1-INH? | |
|---|---|
| **Nur On-Demand-Therapie** | % |
| **C1-INH Substitution i.v.** | % |
| **C1-INH Substitution s.c.** | % |
| **Plasmakallikrein-Inhibitor (Antikörper) s.c.** | % |
| **Plasmakallikrein-Inhibitor (small molecule) p.o.** | % |
| **Antifibrinolytikum p.o.** | % |
| **Sonstige (bitte angeben):** | % |
